# Supplementary material for: Sub-Millisecond Photoinduced Dynamics of Free and EL222-Bound FMN by Stimulated Raman and Visible Absorption Spectroscopies
Source: Biomolecules. 2023 Jan 12;13(1):161. doi: 10.3390/biom13010161 (PMC9855911; doi:10.3390/biom13010161)
Supplement: Supplementary file 1 [file biomolecules-13-00161-s001.zip › biomolecules-2106150-supplementary.pdf]

SUPPLEMENTARY INFORMATION

Sub-millisecond Photoinduced Dynamics of Free and EL222-bound FMN by Stimulated Raman and Visible Absorption Spectroscopies

Yingliang Liu [1,2], Aditya S. Chaudhari [1,3], Aditi Chatterjee [1,3], Prokopis C. Andrikopoulos [1], Alessandra Picchiotti [2,4], Mateusz Rebarz [2], Miroslav Kloz [2], Victor A. Lorenz-Fonfria [5], Bohdan Schneider [1], and Gustavo Fuertes [1,*]

[1] Institute of Biotechnology of the Czech Academy of Sciences, Prumyslova 595, 25250 Vestec, Czech Republic

[2] Extreme Light Infrastructure Beamlines, Institute of Physics of the Czech Academy of Sciences, Za Radnicí 835, 25241 Dolní Břežany, Czech Republic

[3] Faculty of Science, Charles University, Albertov 6, 128 00 Prague, Czech Republic

[4] Hamburg University, Harbor, The Hamburg Centre for Ultrafast Imaging, Universität Hamburg, 149 Luruper Chaussee, 22761 Hamburg, Germany

[5] Institute of Molecular Science, Universitat de València, Carrer del Catedrático José Beltrán Martínez 2, 46980 Paterna, Spain.

* Correspondence to: Gustavo.fuertes@ibt.cas.cz

Table of Contents

Figure S1. Protein quality control 3
Figure S2. Scheme of our broadband dual visible transient absorption (TA)/FSRS set-up 4
Figure S3. Molecular models of the samples used in this study 5
Figure S4. Lifetime density maps of FMN 6
Figure S5. Lifetime density maps of EL222-WT 7
Figure S6. Lifetime density maps of EL222-C78A 8
Figure S7. *D* lifetime distributions in the low frequency Raman region 9
Figure S8. Transient UV/Visible/near-IR spectra 10
Figure S9. Transient Raman spectra 11
Figure S10. Comparison between the *D* lifetime distributions of EL222-WT in D_2O from different vibrational spectroscopies 12
Figure S11. Comparison among the transient spectra obtained in this work and the literature 13-14
Figure S12. Comparison between lifetime distribution analysis (LDA) and global kinetic analysis (GKA) 15
Table S1. Parameters used for the lifetime distribution analyses of FSRS data 16
Table S2. Kinetic isotope effect (KIE) 17
Supplementary references 18

FIGURE S1.

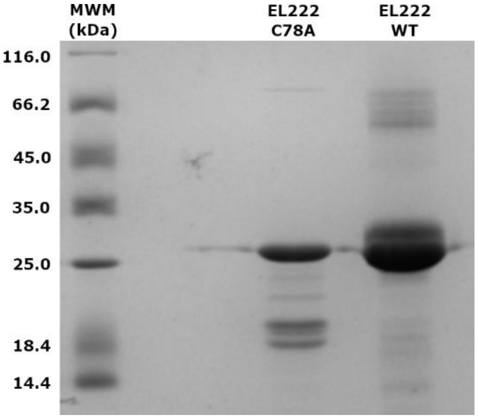

Protein quality control. Coomassie blue stained SDS-PAGE image of purified samples of EL222-WT and EL222-C78A. The masses (in kDa) of the seven proteins constituting the molecular weight marker (MWM) are indicated on the left.

FIGURE S2.

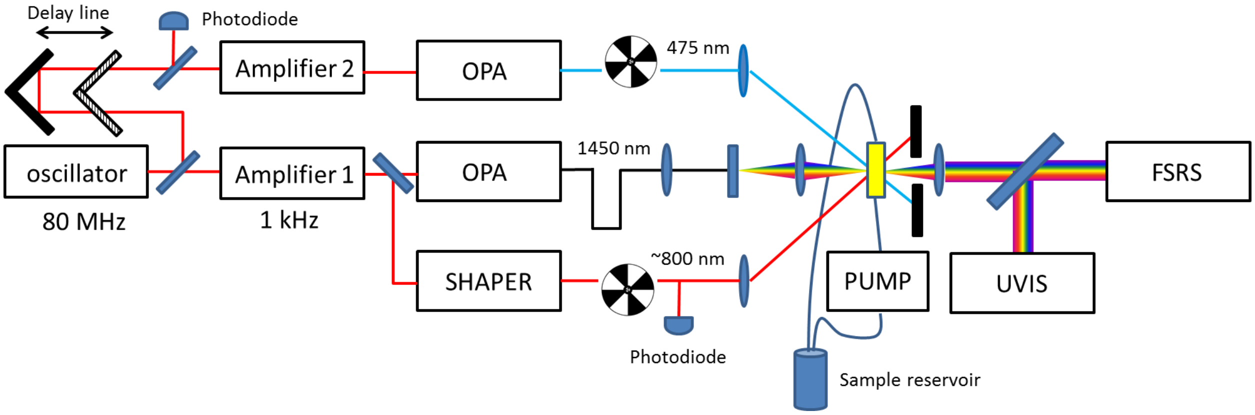

Scheme of our broadband dual visible transient absorption (visTA)/FSRS set-up. The oscillator beam is split in two. The first part is guided directly to amplifier 1 while the second via a delay line into amplifier 2. A photodiode after the delay line generates 80 MHz clock for the amplifier 2 ensuring correct amplification timing independent on seed optical delay (amplifier 1 is clocked directly form the oscillator via BNC cable). This configuration permits to set a seamless time delay between amplifier 1 and amplifier 2 anywhere between 0 fs and ~0.8 ms with accuracy better than 50 fs. The output of amplifier 1 is guided into a commercial optical parameter amplifier (OPA, Light conversion) and converted into 475 nm actinic pulses triggering the reaction. This beam goes via a chopper that reduces its frequency from 1 kHz to 500 Hz. The output of amplifier 1 is split in two parts. The first is pumping an OPA (Light conversion) converting ~800 nm femtosecond (fs) pulses into 1450 nm fs pulses that drive white-light generation in the range from 380 nm to 1200 nm in a calcium fluoride plate enabling Stokes shift from 200 cm-1 to 2200 cm-1. The second is guided through a pulse shaper that includes a modulator chopper blade generating wavelength-modulated spectrally narrowed Raman pulses. Raman and probe pulses are synchronized via a manual delay. All pulses interact with the sample. The probe pulse is removed from the other pulses by an aperture and split in two parts. One goes into FSRS analyzer using optical grating and the other into TA analyzer using prism as a dispersive element. The three beams (actinic, Raman pump, probe) interacts with the sample flowing through a 1 mm thick cell. The total required sample volume (flow cell plus reservoir plus tubing plus dead volume of the pump) is less than 1 mL.

FIGURE S3.

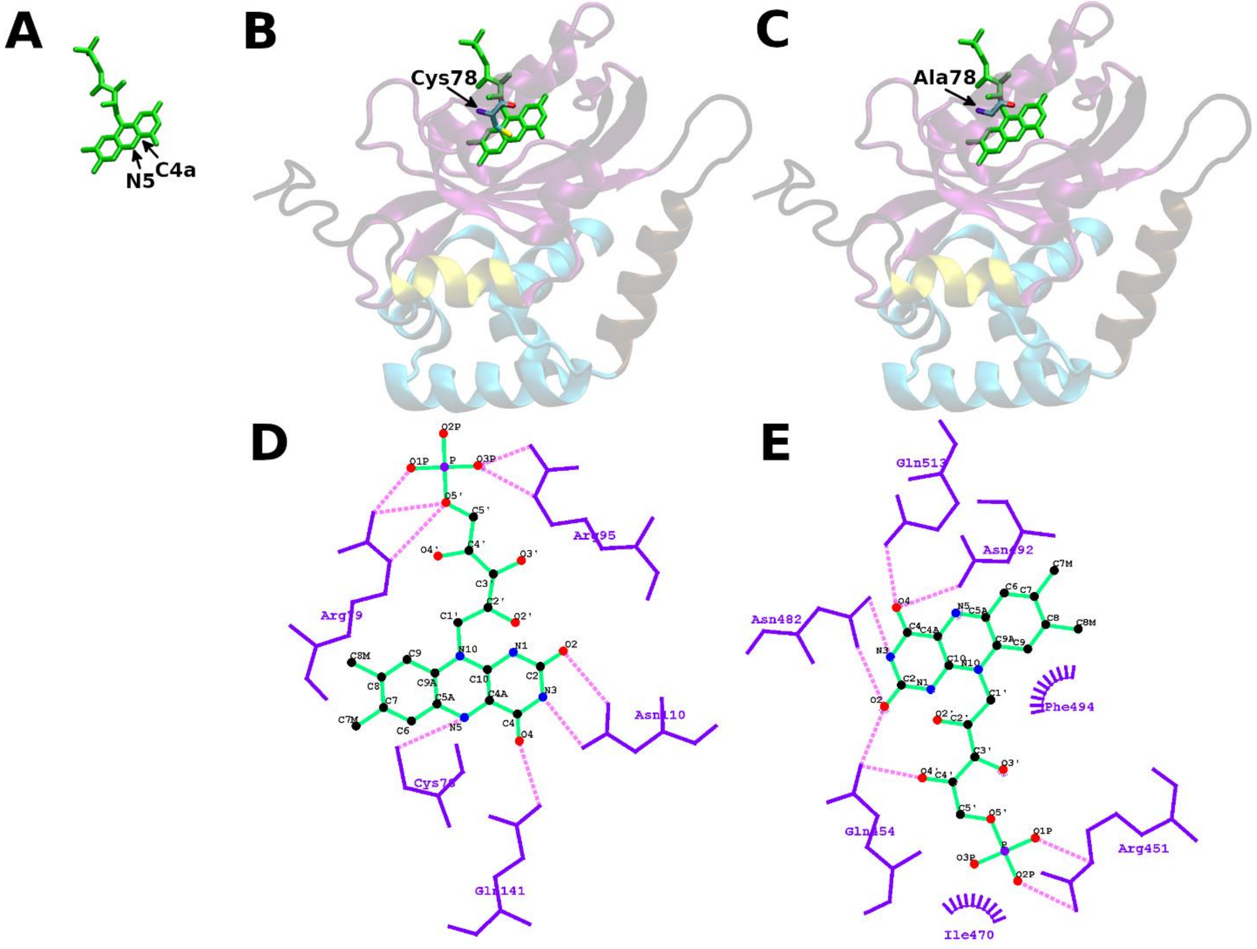

Molecular models of the samples used in this study. (A) FMN. (B) EL222-WT. (C) EL222-C78A. All three structures are based on PDB 3P7N [6] and have been created using Visual Molecular Dynamics software [66]. (D) Scheme of the FMN binding pocket of EL222 (PDB ID 3P7N) [6]. (E) Scheme of the FMN binding pocket of AsLOV2 (PDB ID: 2V0U) [67]. Panels (D) and (E) have been created with LigPlot [68].

FIGURE S4.

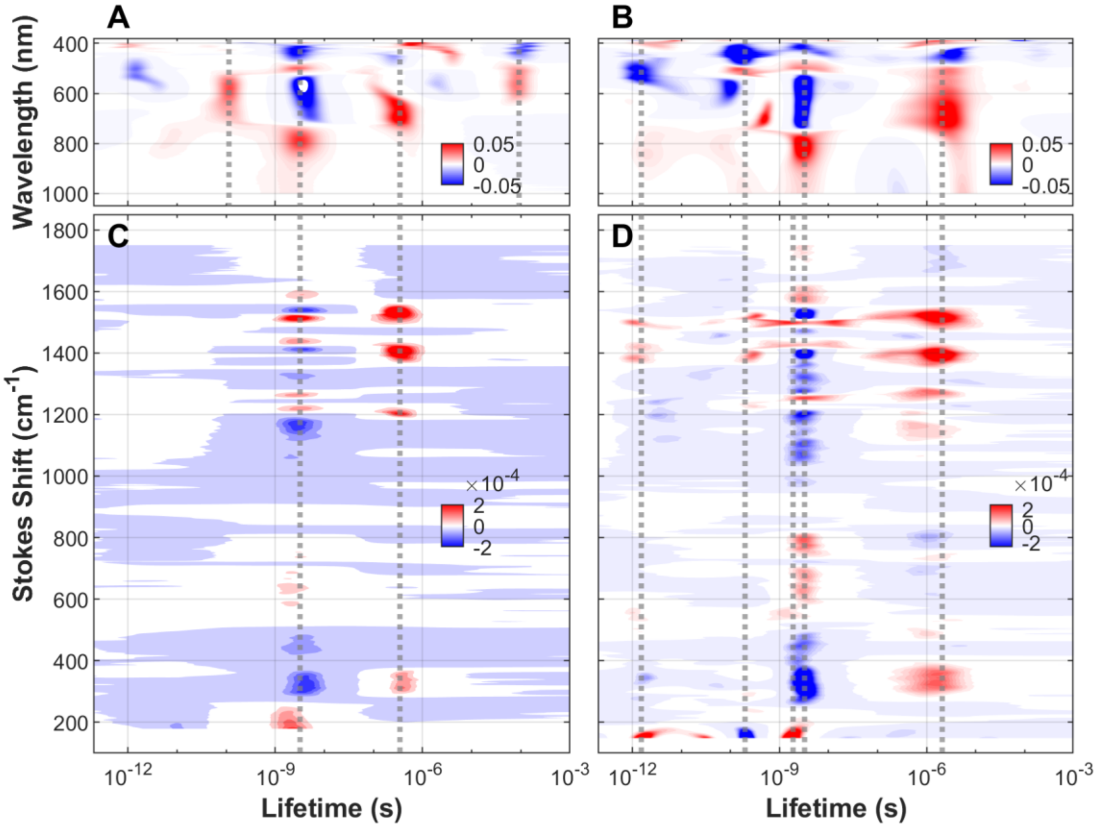

Lifetime density maps of FMN. (A) TA spectroscopy in H_2O-based buffer. (B) TA spectroscopy in D_2O-based buffer. (C) Transient FSRS in H_2O-based buffer. (D) Transient FSRS in D_2O-based buffer. The red color indicates a positive change of the amplitudes (appearing bands). On the contrary, the blue color code indicates a negative change of the amplitudes (disappearing bands). Maps have been calculated by applying the inverse Laplace transform and the maximum entropy method to the corresponding datasets shown in **Figure 1**. Dotted lines indicate the lifetimes of the main dynamical events (values reported in **Table 1**).

FIGURE S5.

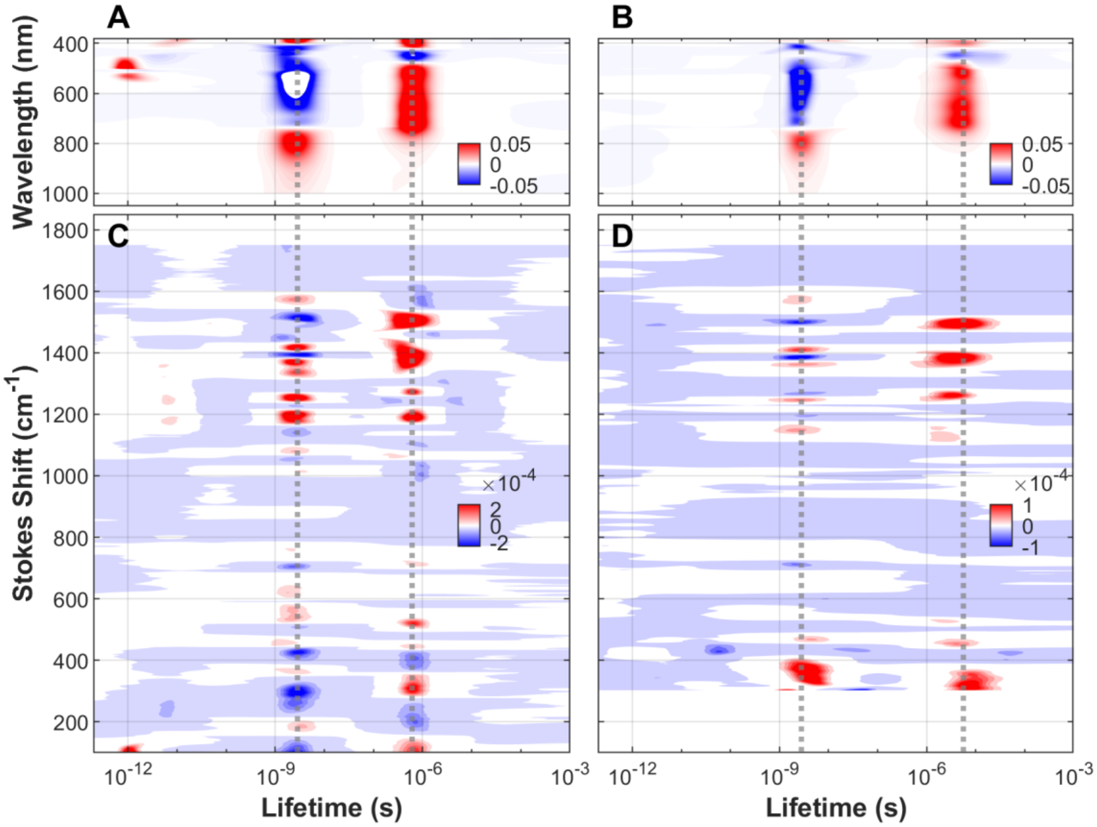

Lifetime density maps of EL222-WT. (A) TA spectroscopy in H_2O-based buffer. (B) TA spectroscopy in D_2O-based buffer. (C) Transient FSRS in H_2O-based buffer. (D) Transient FSRS in D_2O-based buffer. The red color indicates a positive change of the amplitudes (appearing bands). On the contrary, the blue color code indicates a negative change of the amplitudes (disappearing bands). Maps have been calculated by applying the inverse Laplace transform and the maximum entropy method to the corresponding datasets shown in **Figure 2**. Dotted lines indicate the lifetimes of the main dynamical events (values reported in **Table 1**).

FIGURE S6.

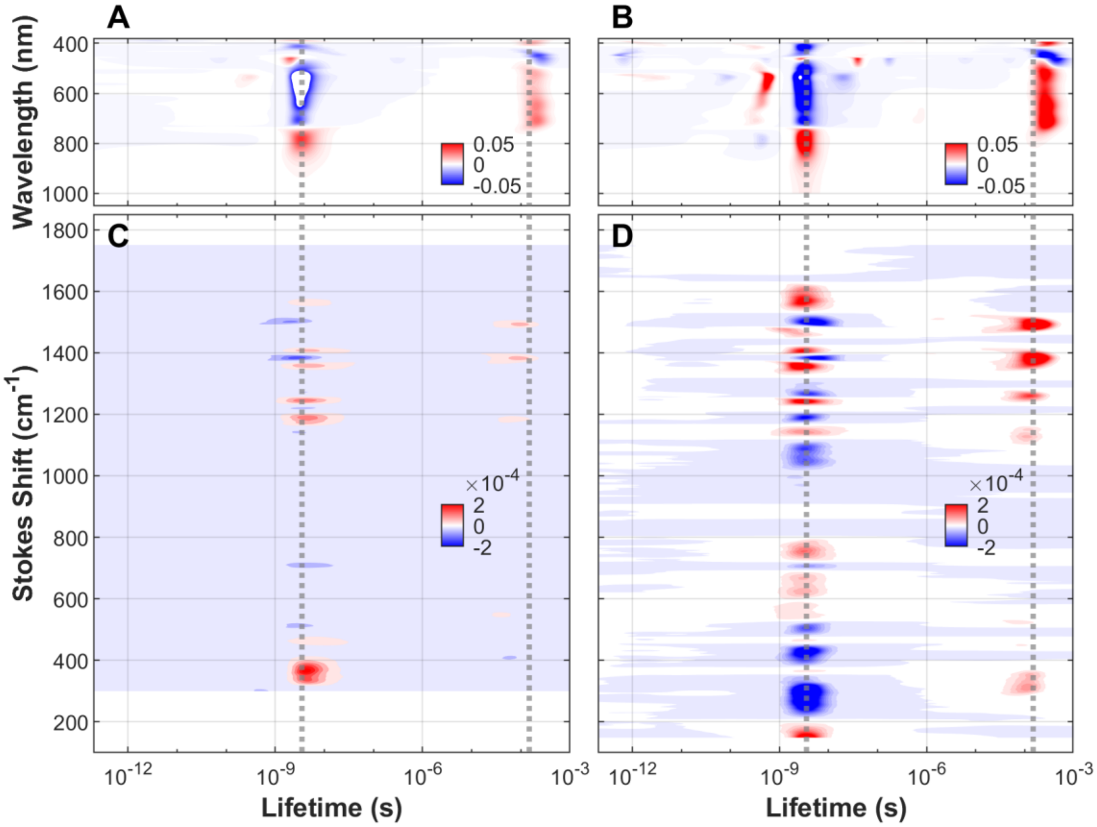

Lifetime density maps of EL222-C78A. (A) TA spectroscopy in H_2O-based buffer. (B) TA spectroscopy in D_2O-based buffer. (C) Transient FSRS in H_2O-based buffer. (D) Transient FSRS in D_2O-based buffer. The red color indicates a positive change of the amplitudes (appearing bands). On the contrary, the blue color code indicates a negative change of the amplitudes (disappearing bands). Maps have been calculated by applying the inverse Laplace transform and the maximum entropy method to the corresponding datasets shown in **Figure 3**. Dotted lines indicate the lifetimes of the main dynamical events (values reported in **Table 1**).

FIGURE S7.

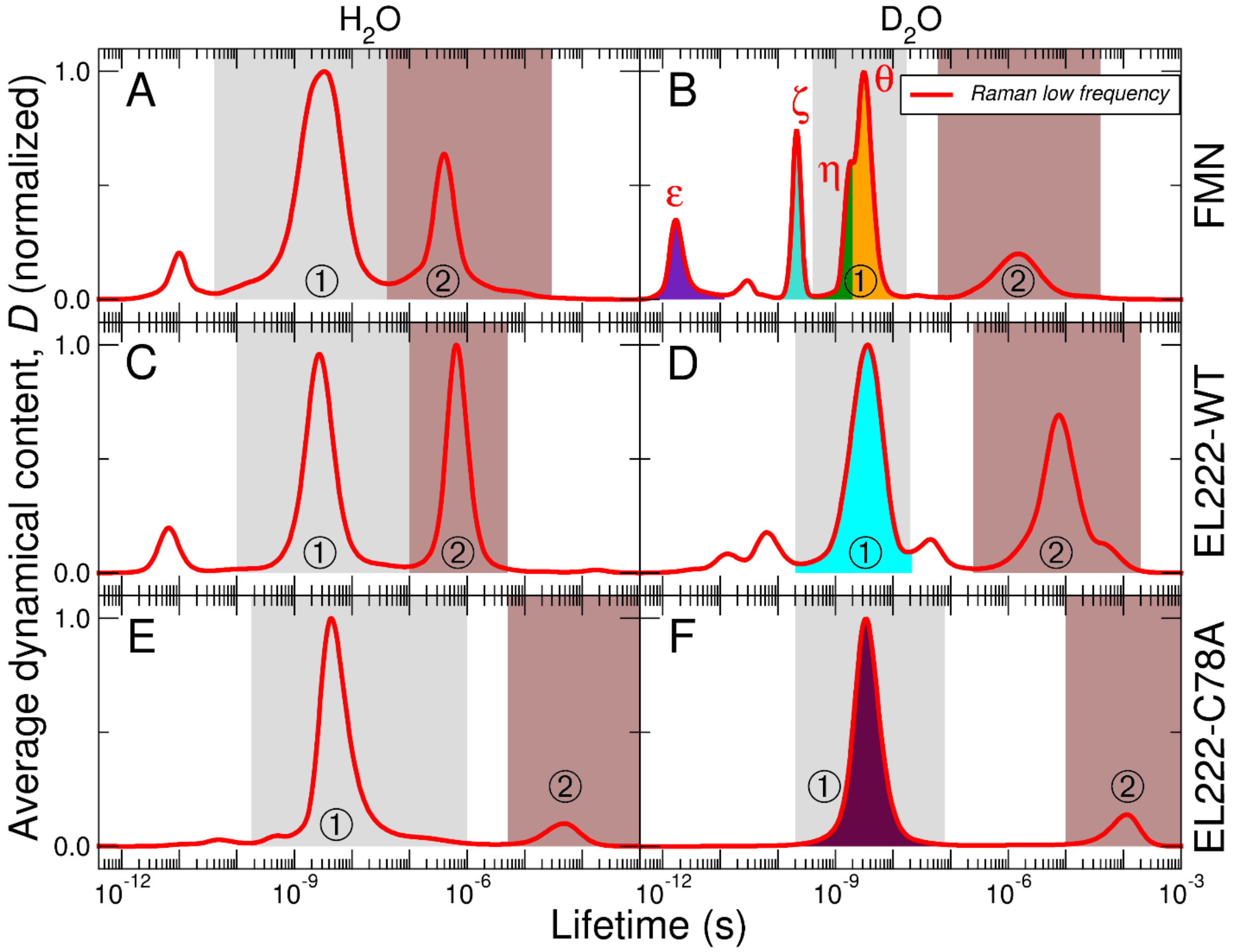

***D* lifetime distributions in the low frequency Raman region.** (A) FMN in H_2O. (B) FMN in D_2O. (C) EL222-WT in H_2O. (D) EL222-WT in D_2O. (E) EL222-C78A in H_2O. (F) EL222-C78A in D_2O. The curves have been calculated from the lifetime density plots shown in **Figure S4**, **S5**, and **S6**, by averaging over the whole Raman low frequency (1000-149 cm^{-1} depending on the dataset, red lines). The two most abundant events present at all probed frequencies are labeled as ① (gray background) and ② (brown background). Extra dynamical events found only in the ~149-1000 cm^{-1} spectral regions with Greek letters (ε, ζ, η, and θ). The exact Raman range analyzed is shown in **Table S1**. The shaded areas in different colors indicate the time scales over which the data sets have been integrated in order to derive the transient spectra shown in **Figure S9**.

FIGURE S8.

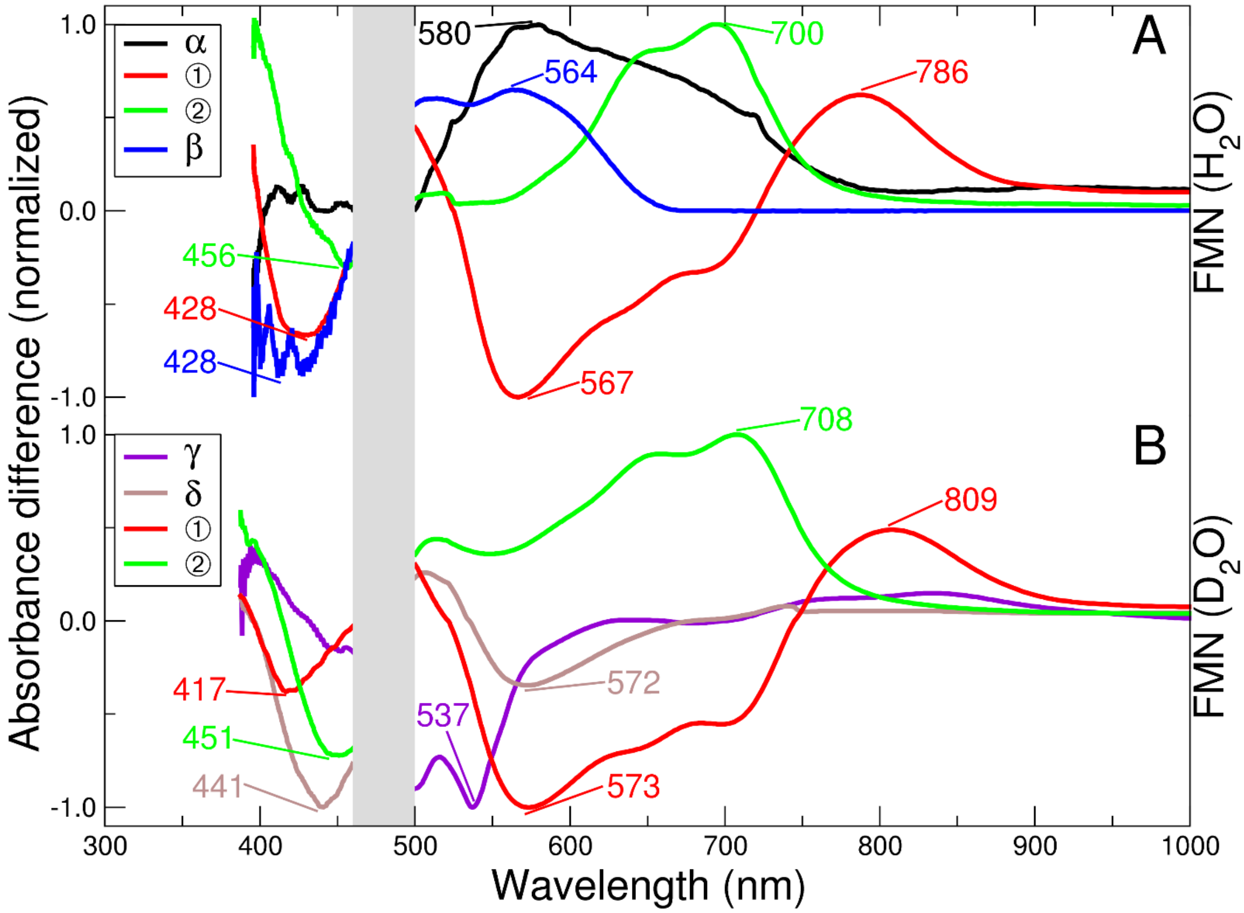

Transient UV/Visible/near-IR spectra. Integrated spectra, equivalent to decay-associated difference spectra (DADS) have been obtained by lifetime distribution analysis using the maximum entropy method. (A) Free FMN in H_2O. (B) Free FMN in D_2O. The wavelength range from 460 to 500 nm (gray area) was excluded from the analysis. The numbers indicate the peak maxima (in nm) of the principal bands.

FIGURE S9.

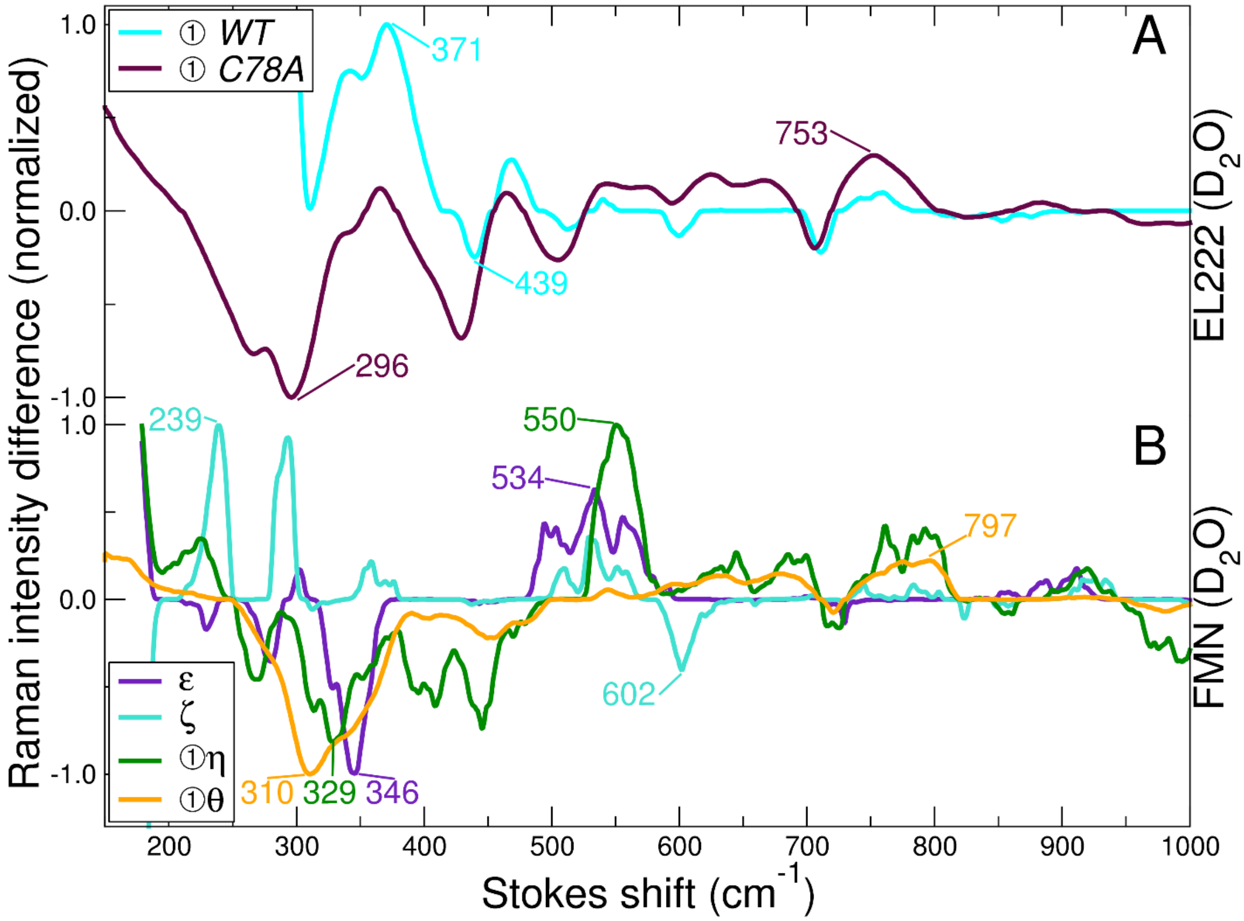

Transient Raman spectra. Integrated spectra, equivalent to decay-associated difference spectra (DADS) have been obtained by lifetime distribution analysis using the maximum entropy method. (A) EL222-WT and EL222-C78A in D_2O. (B) FMN in D_2O. The numbers indicate the peak maxima (in cm^{-1}) of the principal bands.

FIGURE S10.

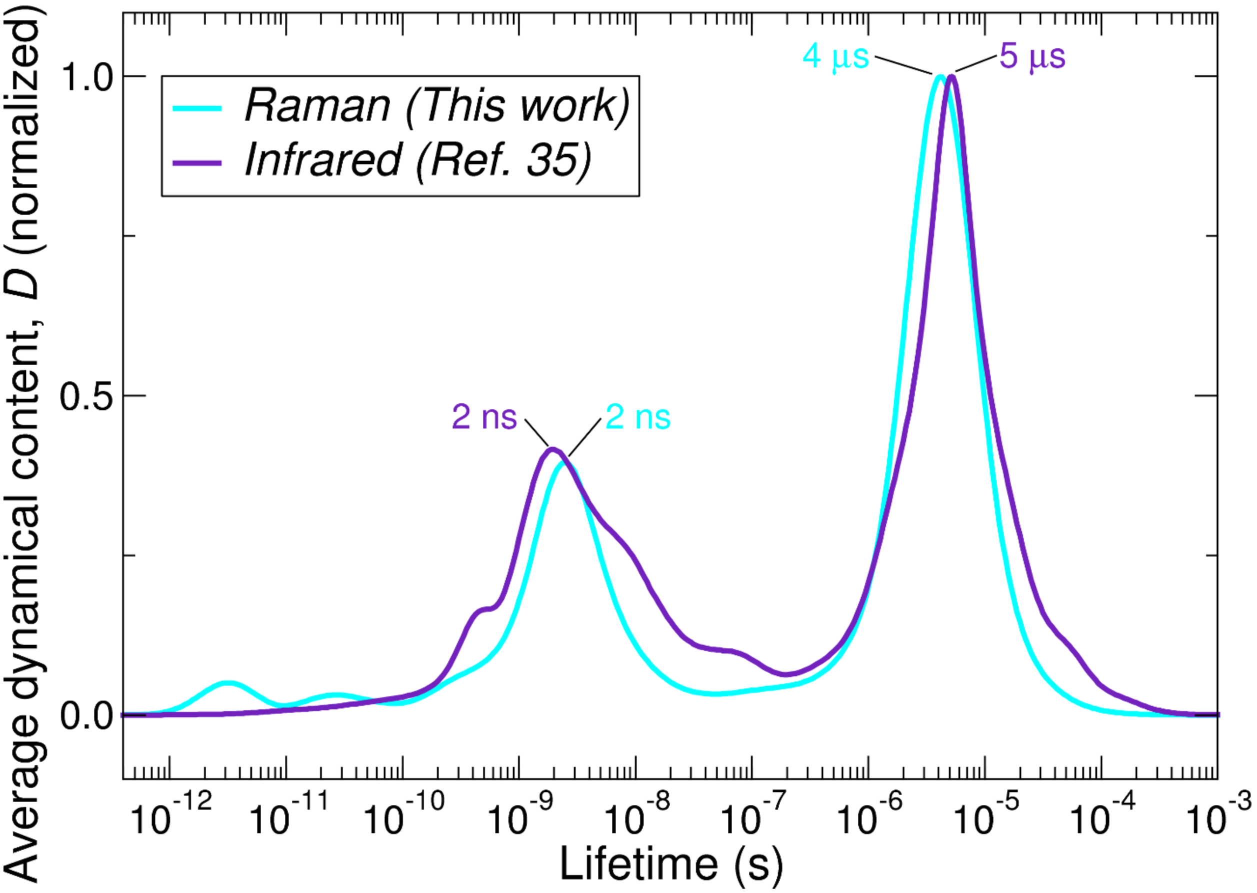

Comparison between the *D* lifetime distributions of EL222-WT in D_2O from different vibrational spectroscopies. Time-resolved Raman (FSRS) data have been taken from this work (Figure 2) while time-resolved infrared data have been taken from our previous work (Reference [35]). Both curves have been calculated by lifetime distribution analysis using the maximum entropy method over the 1500-1750 cm^{-1} frequency region.

FIGURE S11.

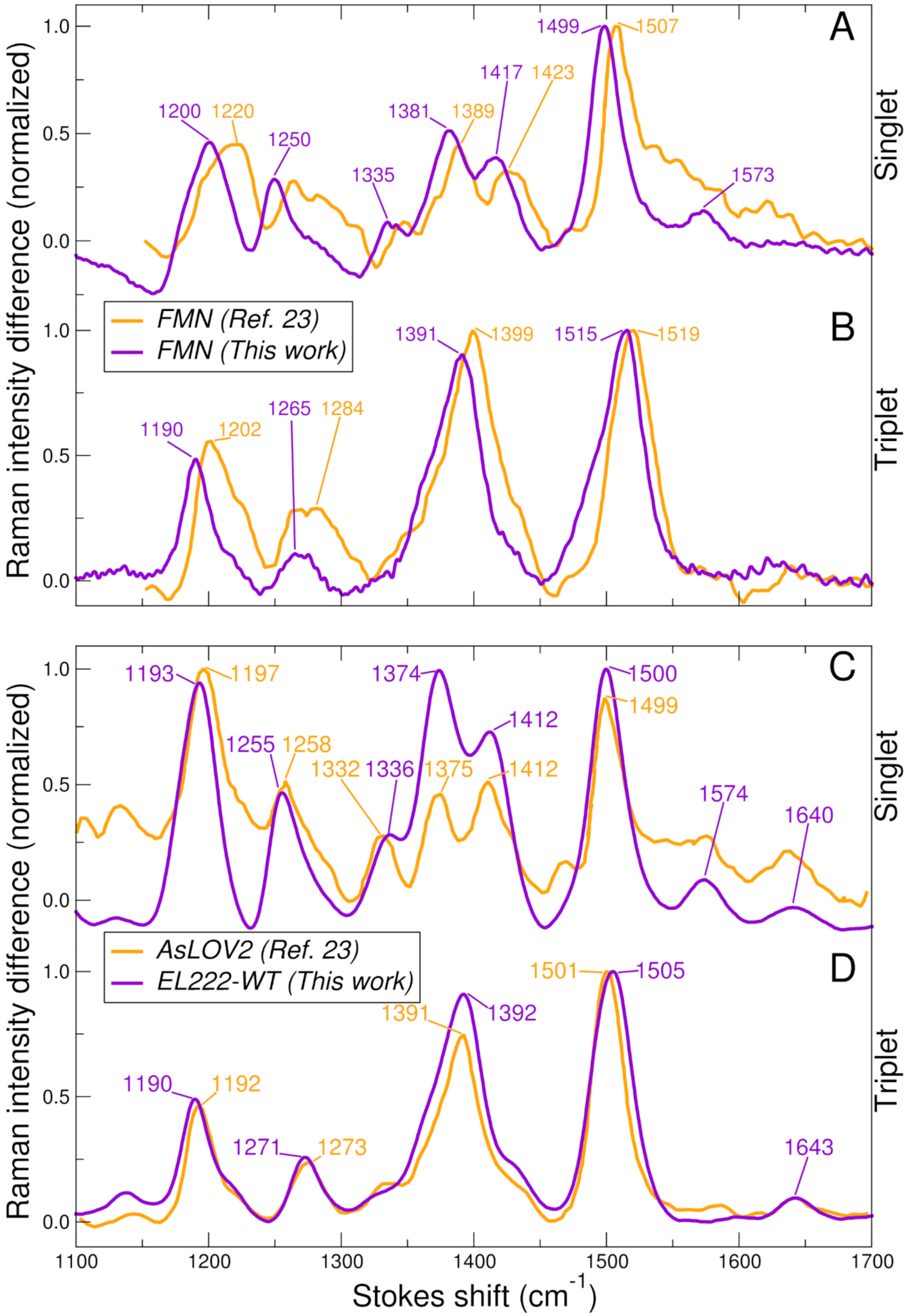

Raman intensity difference (normalized)
Stokes shift (cm^{-1})
A
B
C
D
Singlet
Triplet
Singlet
Triplet
FMN (Ref. 23)
FMN (This work)
AsLOV2 (Ref. 23)
EL222-WT (This work)
1499
1507
1417
1423
1200
1220
1381
1389
1250
1335
1573
1391
1399
1515
1519
1190
1202
1265
1284
1193
1197
1374
1500
1499
1412
1255
1258
1332
1336
1375
1412
1574
1640
1501
1505
1391
1392
1190
1192
1271
1273
1643

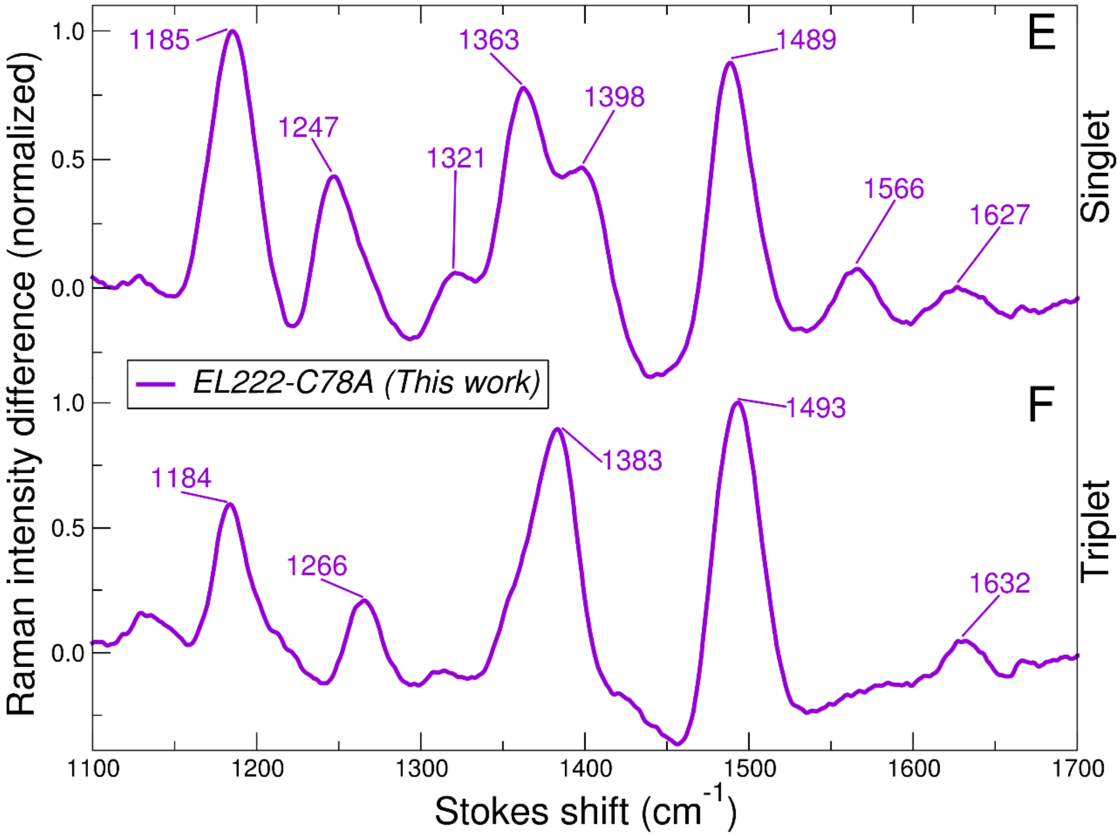

Comparison among the transient spectra obtained in this work and the literature. Evolution-associated difference spectra (EADS) have been obtained by global kinetic analysis using a sequential model. (A) Singlet state of free FMN in H_2O. (B) Triplet state of free FMN in H_2O. (C) Singlet state of WT-bound FMN in H_2O. (D) Triplet state of WT-bound FMN in H_2O. (E) Singlet state of C78A-bound FMN in H_2O. (F) Triplet state of C78A-bound FMN in D_2O. Datasets from this work are shown in violet. Data shown in orange haven been taken from Reference [23]. The numbers indicate the peak maxima (in cm^{-1}) of the principal bands (for AsLOV2 only peaks considered significant in [23] are labeled).

FIGURE S12.

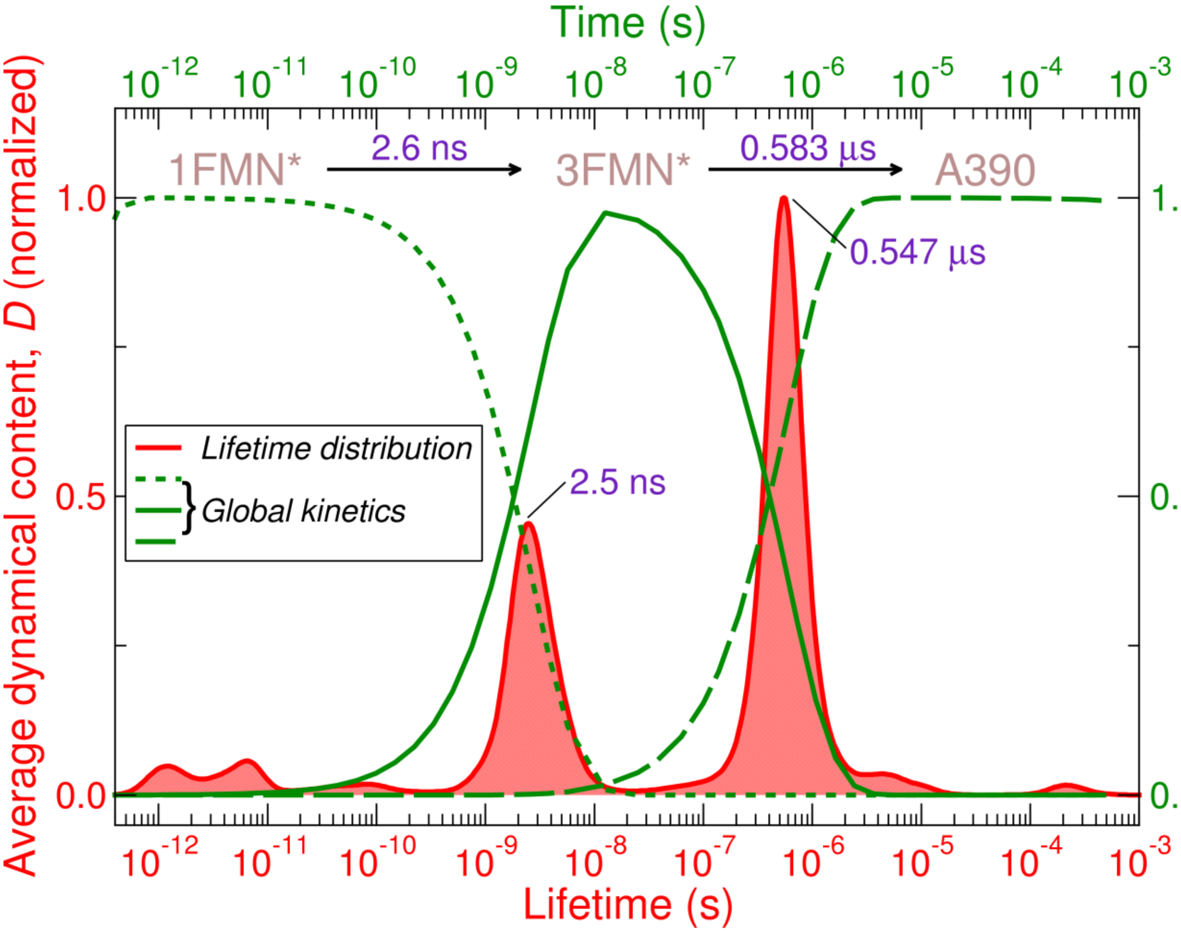

Comparison between lifetime distribution analysis (LDA) and global kinetic analysis (GKA). The data correspond to transient FSRS of EL222-WT in H_2O (high frequency region from 1750 to 1000 cm^{-1}). The *D* lifetime distribution arising from LDA is shown in red. The species fraction as a function of time delay arising from GKA using a two-step sequential model (on top) is shown in green. The retrieved lifetimes are colored in violet.

TABLE S1.

	Solvent	Sample		
		**FMN**	**EL222-WT**	**EL222-C78A**
**Raman shift (cm^{-1})**	**H_2O**	179 (-5.2)	149 (-5.8)	303* (-4.8)
	**D_2O**	149 (-5.5)	303* (-4.9)	149 (-5.2)

(*) The original datasets were collected down to 149 cm^{-1}. However, due to noise in the time domain, Stokes shifts in the range 149-302 cm^{-1} were excluded.

Parameters used for the lifetime distribution analyses of FSRS data. The lowest measured frequency values are indicated. Due to differences in the measured ranges, the low frequency Raman region was analyzed twice: from 303 to 1000 cm^{-1} (**Figure 4**), i.e. same data range for all sets, or from reported values to 1000 cm^{-1} (**Figure S7**), i.e. distinct range for each dataset. The optimum regularization parameters (λ_{opt}), given as $\log_{10}(\lambda_{opt})$, according to the L-curve criterion are shown in parenthesis.

TABLE S2.

FMN			EL222-WT			EL222-C78A			Sample	
**RH**	**RL**	**TA**	**RH**	**RL**	**TA**	**RH**	**RL**	**TA**	**Scale**	**Dynamical event**
1.1	0.9	0.8	1.0	1.4	1.0	0.8	0.8	0.8	ns	① in Figure 4
5.4	3.8	9.0	7.6	11.5	7.8	1.3		1.3	μs	② in Figure 4

Kinetic isotope effect (KIE) calculated by dividing the lifetimes in D_2O by the lifetimes in H_2O. Only the two main dynamical events shown in Figure 4 were considered. RH = Raman high frequency region (1750-1000-cm^{-1}, blue color). RL=Raman low frequency region (1000-303 cm^{-1}, red color), TA=transient absorption (380-1000 nm, green color).

Supplementary references.

66. Humphrey, W.; Dalke, A.; Schulten, K. VMD: Visual molecular dynamics. Journal of Molecular Graphics 1996, 14, 33-38, doi:10.1016/0263-7855(96)00018-5.

67. Halavaty, A.S.; Moffat, K. N- and C-Terminal Flanking Regions Modulate Light-Induced Signal Transduction in the LOV2 Domain of the Blue Light Sensor Phototropin 1 from Avena sativa. Biochemistry 2007, 46, 14001-14009, doi:10.1021/bi701543e.

68. Wallace, A.C.; Laskowski, R.A.; Thornton, J.M. LIGPLOT: a program to generate schematic diagrams of protein-ligand interactions. "Protein Engineering, Design and Selection" 1995, 8, 127-134, doi:10.1093/protein/8.2.127
